# Considerations for Designing Disability-Inclusive Music Programs for Older Adults

**DOI:** 10.1093/geroni/igaf122.3350

**Published:** 2025-12-31

**Authors:** Jennie Dorris, Lauren Terhorst, Juleen Rodakowski

**Affiliations:** University of Pittsburgh, Pittsburgh, Pennsylvania, United States; University of Pittsburgh, Pittsburgh, Pennsylvania, United States; University of Pittsburgh, Pittsburgh, Pennsylvania, United States

## Abstract

Music is a popular modality that has shown promise to support healthy adults and those living with disabilities. As adults age, they are more likely to report a disability and experience health disparities.

**Purpose:**

The objective of our exploratory study was to create an understanding of disability and music participation for adults over 65 years old.

**Materials and methods:**

We conducted a secondary data analysis of survey data from the 2022 Current Population Survey, administered to homes in the community in the United States. We included adults over 65 years of age. Disability was measured by a self-reported yes/no response. Music participation was measured by a self-reported yes/no response to either singing or playing a musical instrument in the last 12 months.

**Results:**

In a simple logistic regression and a model controlling for demographic characteristics, having a disability was not statistically significantly associated with music participation, β = 0.0351, p = 0.735, (OR = 1.073, 95% CI [0.714, 1.611]).

**Conclusions:**

These findings lay the groundwork for future Stage 1 research to design music interventions with universal inclusion of those with and without disabilities to maximize music’s potential impacts on older adults.

